# Remote Lifestyle Counseling Influences Cardiovascular Health Outcomes in Youth with Overweight or Obesity and Congenital Heart Disease

**DOI:** 10.3389/fped.2017.00269

**Published:** 2017-12-18

**Authors:** Luis Altamirano-Diaz, Meghan Rombeek, Stefanie De Jesus, Eva Welisch, Harry Prapavessis, Adam A. Dempsey, Douglas Fraser, Michael R. Miller, Kambiz Norozi

**Affiliations:** ^1^Department of Paediatrics, Western University, London, ON, Canada; ^2^Paediatric Cardiopulmonary Research Laboratory, London Health Science Centre, London, ON, Canada; ^3^Children’s Health Research Institute, London, ON, Canada; ^4^School of Kinesiology, Western University, London, ON, Canada; ^5^Translational Research Centre, London, ON, Canada; ^6^Lawson Health Research Institute, London, ON, Canada; ^7^Department of Paediatric Cardiology and Intensive Care Medicine, Medical School Hannover, Hannover, Germany; ^8^Department of Paediatric Cardiology and Intensive Care Medicine, University of Goettingen, Goettingen, Germany

**Keywords:** smartphone, congenital heart disease, remote counseling, obesity, children, lifestyle intervention

## Abstract

**Background:**

Children with overweight/obesity and congenital heart disease (CHD) are at increased cardiovascular risk. A lifestyle intervention may help reduce these risks. We sought to determine the feasibility of a smartphone-based lifestyle intervention to improve cardiovascular health outcomes in children with overweight/obesity and CHD.

**Methods:**

We examined the effect of bi-weekly nutrition and fitness counseling delivered *via* smartphone over 12 months. Thirty-four youth, previously diagnosed with CHD and with overweight or obesity, participated in the intervention. They were divided into two groups depending on whether the heart disease required surgical correction (operated, *n* = 19) or not (non-operated, *n* = 15). Anthropometry, body composition cardiorespiratory exercise capacity, and cardio-metabolic risk factors were assessed at baseline, 6 months, and 12 months.

**Results:**

Statistically significant decreases in waist circumference (WC), body mass index *z*-score, WC *z*-score, and waist to height ratio *z*-score were observed at 6 and 12 months in the operated group. A significant linear increase in lean body mass was observed in both groups. The study also had a high retention rate and a low attrition rate.

**Conclusion:**

The observed changes in anthropometry were positive with significant improvement to some cardiovascular and metabolic risk indicators. However, this was only observed in the operated group suggesting that other factors, such as perception of condition and self-efficacy, may influence lifestyle behaviors. The results from this pilot study clearly demonstrate the feasibility to perform a larger controlled study on remote lifestyle intervention in children with congenital heart defects and overweight or obesity.

## Introduction

Currently, about one-third of children in Canada and the United States are overweight or obese, and our group and others have found that the prevalence of overweight and obesity between children with congenital heart disease (CHD) and healthy children do not differ significantly (1–3). The numerous cardiovascular risks, and physical and psychosocial health consequences of childhood obesity are well reviewed (4, 5). Although evidence is now emerging, children with overweight or obesity and CHD appear more likely to exhibit additional cardiovascular risk factors, such as higher systolic blood pressure and lower high density lipoproteins (HDL), compared to children of normal weight (1, 6).

Unhealthy eating habits, sedentary behavior, and reduced physical activity can increase the risk of obesity and cardiovascular disease (7). Children with CHD are less physically active (8) and have a lower health-related quality of life than their peers (9). Although a structured lifestyle intervention for children and youth with overweight or obesity and CHD has never been completed, it has the potential to diminish cardiovascular health risks by improving nutrition, physical fitness, body composition, and related health outcomes (10). However, conventional pediatric lifestyle intervention programs struggle with barriers to their success, such as high attrition rates and therapeutic non-compliance (11).

Smartphones may address some of the inherent challenges of structured lifestyle interventions by eliminating geographical barriers, maintaining the home environment and appealing to a more technologically savvy generation. Overall, mobile devices offer promising results for improving weight loss and health behaviors, but their application has not been explored in a pediatric population with overweight or obesity and CHD (12–14). In addition, weight-loss interventions using remote counseling have been shown to be as effective as in-person support for weight loss in an adult population with obesity (15).

The objective of the “Smart Heart” Pilot Study (registered as NCT02980393 at http://www.ClinicalTrials.gov) was to examine the cardiovascular health impact of a 12-month, structured lifestyle intervention program for children and adolescents with overweight or obesity and CHD through the use of smartphones. It was hypothesized that participants would demonstrate favorable changes in anthropometric, body composition, exercise capacity, and metabolic parameters associated with cardiovascular health. We also sought to determine if self-efficacy resulting from the perceived severity of CHD influences the outcomes of the intervention.

## Materials and Methods

### Study Design

Eligible candidates were selected during routine visits and through chart review by cardiologists at the London Health Sciences Centre (LHSC) (London, ON, Canada). Consenting individuals underwent a physical assessment and a review of medications and comorbidities during the first stage of measurements. All patients were recruited from within our catchment area of Southwestern Ontario, Canada.

Two groups of patients were recruited for this study, those who had undergone corrective surgery for CHD (“operated”) and those with minor heart defects that do not require surgical correction (“non-operated”). All subjects had overweight or obesity. Both groups received the identical intervention.

Candidates were excluded from the study if they: had severe residual heart disease, were at risk for a worsening cardiac condition, were unable to participate due to mental or physical disabilities, had imposed exercise restrictions, were taking confounding medications, had comorbidities affecting weight or metabolic conditions, or were involved in any concurrent lifestyle intervention. This study was approved by the Western University Health Science Research Ethics Board (REB#18843) and the Lawson Health Research Institute (R-12-266). All subjects gave written informed consent in accordance with the Declaration of Helsinki. All children and adolescents gave written informed consent to participate in this study. For those under 16 years of age, we obtained written informed consent from their parents/guardians.

**Figure 1:**
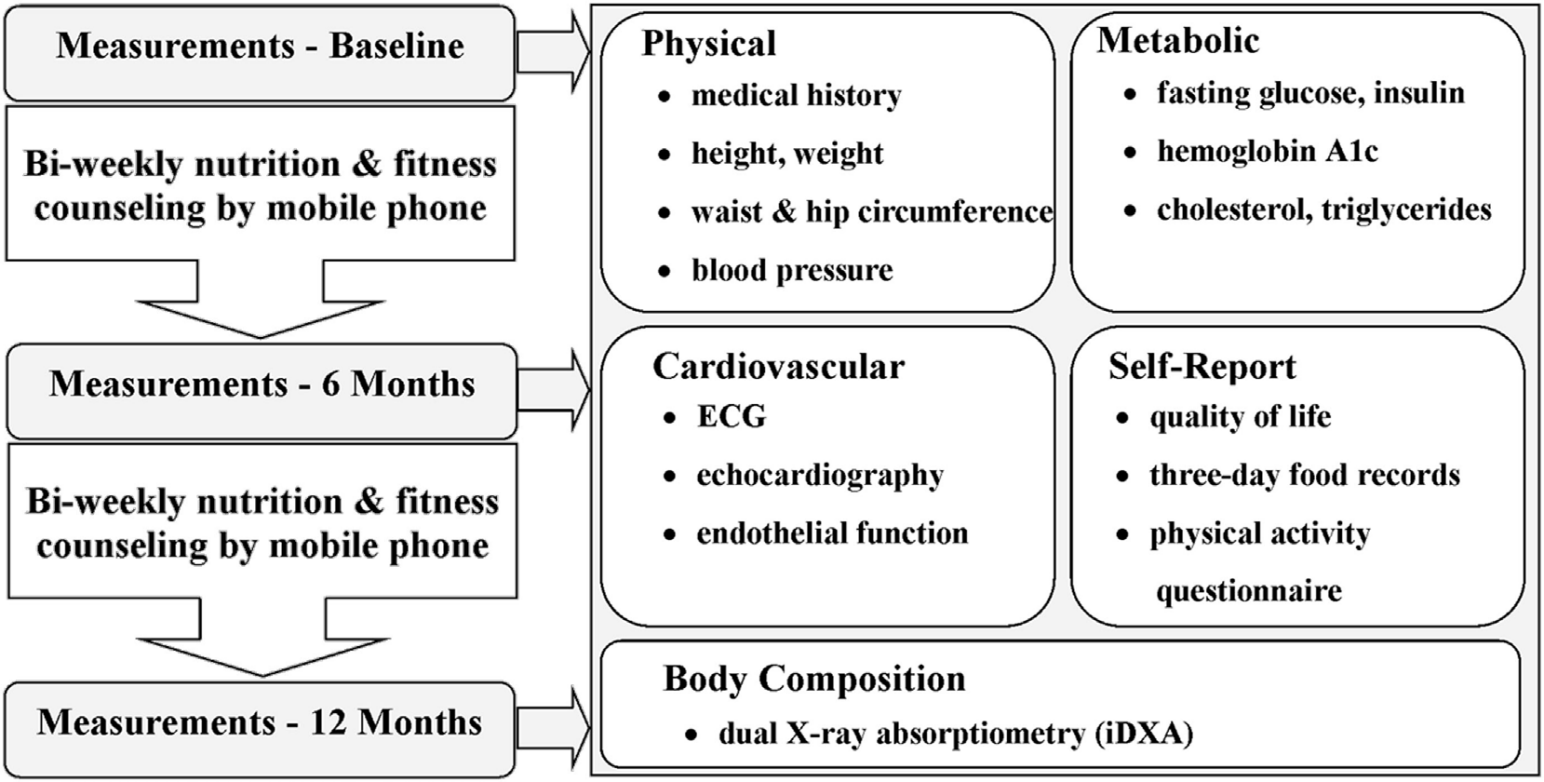
Flow diagram of the Smart Heart Pilot Study procedure from baseline to 12-month measures. The Smart Heart Pilot Study included three assessments and spanned 12 months. Self-report, physical, metabolic, cardiovascular, and body composition outcome measures were collected for each participant at baseline and at 6- and 12-month follow-ups. Nutrition and fitness counseling was performed by smartphone once per week, with the nutrition and fitness counseling support alternating weeks (i.e., 25 counseling sessions for each, for a total of 50 sessions).

The primary outcome measures were anthropometry, body composition, and cardiorespiratory exercise capacity. The secondary metrics included biochemical markers associated with cardiovascular health and risk. Outcomes were assessed at baseline and 6 and 12 months following baseline at the Children’s Hospital, LHSC and Western University, Ontario, Canada. The Smart Heart Pilot Study protocol is outlined in Figure 1.

### Primary  Outcome Measures

#### Anthropometry

Weight and height were measured to the nearest 0.1 kg and to the nearest 0.1 cm, respectively. Waist circumference (WC) was measured according to the NHANES III Body Measurements protocol (16). Briefly, WC was measured at least twice with a flexible tape to the nearest 0.1 cm at the tip of the iliac crest. An average was taken of the first two measures with internal variation of ≤1 cm. The *z*-scores were calculated using the following formula: $z=\frac{\left({\left(\frac{X}{M}\right)}^{L}\right)-1}{\mathrm{LS}}$, where *X* is the BMI, WC, or waist to height ratio (WHtR)*; L* is lambda, *M* is mu, and *S* is sigma. Age and gender based values for *L, M*, and *S* were obtained from the growth charts provided by the CDC and Sharma et al. (17, 18).

#### Body Composition Measurements

Dual X-ray absorptiometry (iDXA; General Electric-Lunar iDXA, Ames Medical iDXA; Prodigy, enCORE 2007 software version 11.40.004, Waukesha, WI, USA) was used to measure body fat (kg) and lean body mass (LBM) (kg). Lunar iDXA has been previously validated (19).

#### Cardiorespiratory Exercise Capacity

Tests were performed at the Pediatric Cardiopulmonary Research Laboratory, Children’s Hospital, LHSC, or at the Exercise and Health Psychology Lab, Western University, Ontario, Canada. Breath-by-breath data on the volume of oxygen uptake (VO_2_) (mL) and carbon dioxide production (mL) were collected during a maximal exercise graded treadmill test using a Cosmed Quark b^2^ indirect calorimetry metabolic system (Cosmed S.r.I, Rome, Italy). An electrocardiogram was used simultaneously to monitor heart rate and identify arrhythmias and ST changes. The goal was to determine peak VO_2_ based on a respiratory exchange ratio (*R*) ≥1.05 (20).

### Secondary Outcome Measures

Participants fasted for ≥10 h and blood collected. The lipid profile [total cholesterol, triglycerides and high and low density lipoproteins (HDL, LDL)], fasting plasma glucose, hemoglobin A1C, and insulin levels were measured using standard protocols at the LHSC Core laboratory. The homeostatic model assessment—insulin resistance [homeostatic model assessment for insulin resistance (HOMA-IR)] was calculated using the following formula: ${\text{HOMA}}_{\text{IR}}=\frac{\text{FPG}*(\text{FPI}*0.144)}{22.5}$; where FPG is fasting plasma glucose levels (mmol/L) and FPI is fasting plasma insulin levels (pmol/L).

### Lifestyle Intervention

During the baseline visit, participants were provided a complimentary smartphone. The lifestyle intervention involved alternating weekly phone calls with two health coaches: a registered dietitian and a fitness specialist. A total of 50 phone calls were delivered to each participant over the 12 months. Counseling sessions were ≤30 min. Counseling strategies were focused on education, behaviors, and family engagement and included regular evaluations. The primary counseling topics are outlined in Table 1. The counseling was tailored to the specific needs of each patient (e.g., lactose intolerance, sports nutrition, exercise modifications during injury recovery), and good sleeping habits were emphasized.

**Table 1:** Lifestyle counseling: the primary topics for nutrition and fitness.

**Nutrition program counseling topics** *Education and strategies* Canada’s Food Guide as a resource Planning balanced and healthier meals Approaches to grocery shopping Understanding nutrition labels Choosing healthier drinks over sugar-sweetened drinks Increasing consumption of vegetables and fruit Strategies for eating outside of home Packing lunches Family meals Eating breakfast *Behaviors* Eating in moderation Slower eating Focused eating Division of responsibility in feeding Snacking Hunger and satiety cues Emotional eating *Family engagement* *Phase 1. Months 0–6*: Bi-weekly resources *Phase 2. Months 6–12*: Monthly nutrition challenges *Evaluation* Every 3 months (food records or diet recall) Eating habits questionnaires (4 in total) (*phase 2. Months 6–12 only*)	**Fitness program counseling topics** *Education and strategies* Canada’s Physical Activity and Sedentary Behavior Guidelines as a resource Benefits of physical activity and impacts of sedentary behavior Interconnection of lifestyle behaviors (sleep, activity) Supports and environments (home, school, etc.) Intensity levels *Behaviors* *Phase 1. Months 0–6* Planning and goal-setting Self-monitoring Overcoming barriers Incorporating activity into regular daily routine (e.g., taking the stairs) *Phase 2. Months 6–12* Challenging current physical activity levels and diversity of movement choices Sustaining motivation levels and incorporation of rewards *Evaluation*: Bi-weekly (physical activity recall)

### Statistical Analyses

Means and SDs were calculated for all continuous variables. With the exception of *z*-scores, RM-ANCOVA was conducted for each outcome across the three time points (baseline, 6 months, and 12 months), while adjusting for age and sex. The *z*-scores (already adjusted for age and sex) for BMI (BMI-Z), WC (WC-Z), and WHtR (WHtR-Z) were analyzed using RM-ANOVA. The analyses were performed independently on both the operated and non-operated groups. For outcomes significant at *p* < 0.05, *Bonferroni post hoc* comparisons examined pairwise differences between time points.

**Figure 2:**
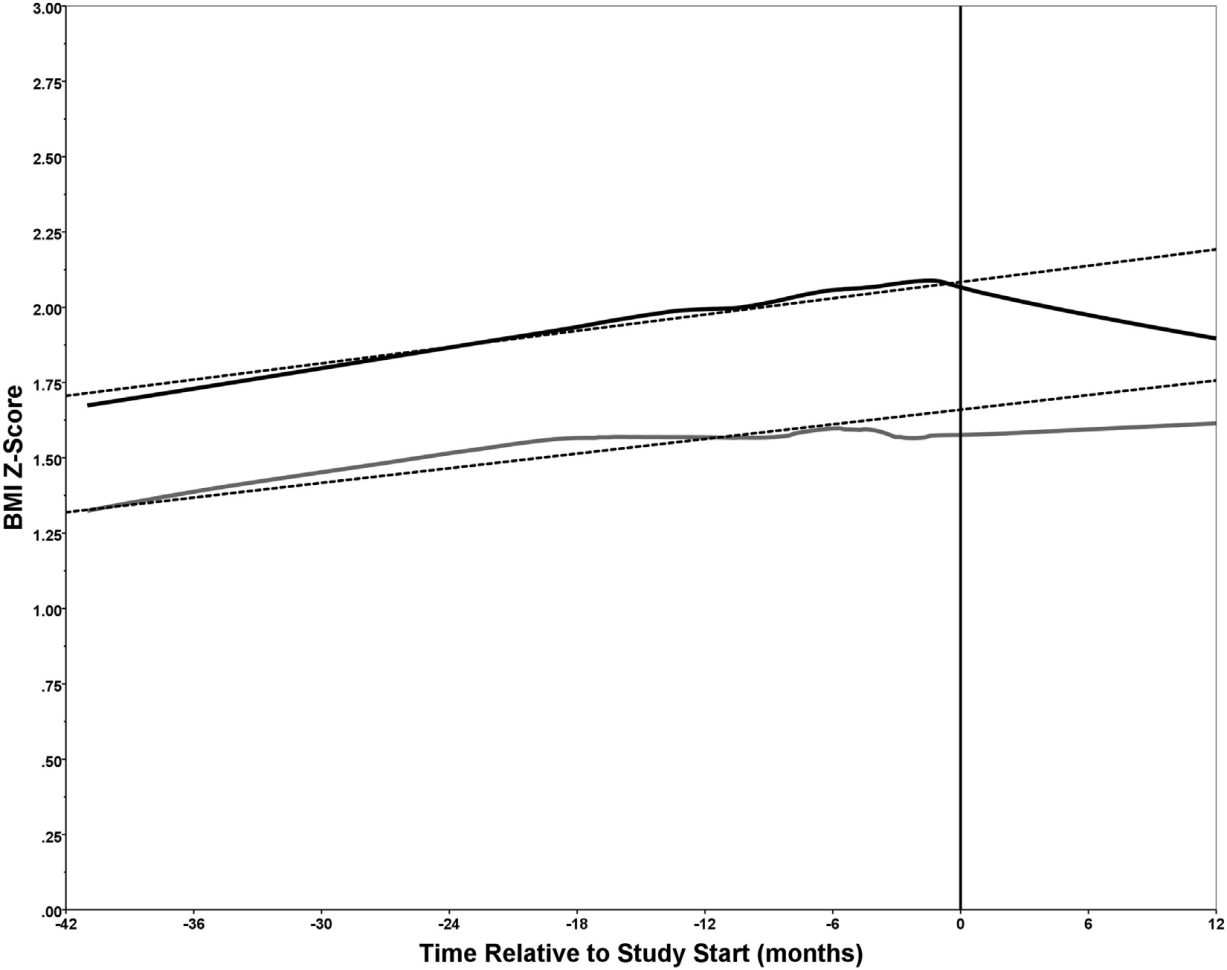
Trends in body mass index *z*-score (BMI-Z) score. The BMI-Z was plotted over time (months) relative to the study start date (month 0) for each of the 34 participants divided into the operated congenital heart disease (CHD) (*n* = 19) and non-operated CHD (*n* = 15) groups. The BMI-Z was determined for each of the 34 study participants, when possible, from 42 months to 6 months prior to the study start date (month 0) and during the intervention at 0, 6, and 12 months. Loess was used to generate a smoothed line of fit for the operated (solid black line) and non-operated (solid gray line) groups. Linear regression was performed using data from months −42 to 0 to determine a trajectory for the BMI-Z (dashed lines). For the operated group, *n* = 14 of 19 had from 2 to 4 BMI-Z measurements prior to the study start; while for the non-operated group, *n* = 11 of 15 had 1 to 3 BMI-Z measurements prior to the study start. All 34 participants had BMI-Z measurements over the course of the intervention (i.e., at 0, 6, and 12 months) with the exception of one participant in each of the two groups that missed the 6-month follow-up. The study start point (0 months) is indicated by a vertical line.

BMI *z*-scores were also calculated from clinic visits from 6 to 42 months prior to the start of the study (Figure 2). This time frame was selected as it represented a long enough period prior to study start to observe any trends and maximized the availability of data for performing linear regression. A smoothed line of fit was determined for each group using locally weighted scatterplot smoothing (loess) and 70% of the data points with the *Epanechnikov* kernel. Linear regression was used to determine the pre-intervention body mass index *z*-score (BMI-Z) trajectory for each group using data up to, and including, the baseline.

All statistical analyses were performed using SPSS v.23 or v.24 (IBM Corporation, Armonk, NY, USA).

## Results

Patient recruitment spanned May 2012 to October 2015. Thirty-four patients (15 females, 19 males) with overweight or obesity (21), between 7 and 17 years of age (mean 14.3 ± 2.8 years) at the time of screening, were recruited. Of these, *n* = 19 had CHD that required corrective surgery (operated group) while *n* = 15 had CHD that did not require corrective surgery (non-operated group). Patient assessments spanned January 2013 to October 2016. One participant from each group did not complete the 6-month follow-up assessment and were excluded from all repeated measures statistical analyses.

The operated group consisted of patients diagnosed with one or more of the following: aortic stenosis, atrial septal defect (type II), atrio-ventricular septal defect, coarctation of the aorta, hemitruncus, hypoplastic left heart syndrome, patent ductus arteriosus, pulmonic stenosis, transposition of the great arteries, or tetralogy of Fallot. While the non-operated group consisted of patients diagnosed with one or more of the following: aortic root dilation, aortic stenosis, aortic valve regurgitation, ascending aortic dilatation, atrial septal defect (type II), bicuspid aortic valve, coronary sinus atresia, coronary sinus draining into innominate vein, mitral valve prolapse, mitral valve regurgitation, patent ductus arteriosus, pulmonic stenosis, subaortic ridge, systolic ejection murmur, or ventricular septal defect.

The fitness and nutrition counselors allocated 30 min for each session: 15–20 min for counseling and 10–15 min for charting. Thus, the primary cost of applying the intervention was approximately a combined 25 professional working hours for the weekly remote smartphone counseling per participant over 12 months.

There was no significant difference between HDL, LDL, TG, fasting plasma glucose, hemoglobin A1C, insulin levels, HOMA-IR, and maximal volume oxygen uptake at baseline, 6 months, or 12 months for both groups.

Tables 2 and 3 summarize the key outcome measures at baseline, 6 months, and 12 months. There was a significant linear decrease in WC, BMI-Z, waist circumference *z*-score (WC-Z), and WHtR-Z over 12 months in the operated group. *Post hoc* comparisons showed significant differences between baseline and both the 6- and 12-month time points for BMI-Z, WC-Z, WHtR-Z, and LBM. There was only a significant difference between baseline and the 6-month time point for WC. By the end of the study, BMI-Z decreased by 0.12 (95% CI 0.002 to 0.28), WC-Z decreased by 0.24 (95% CI 0.05 to 0.43), and WHtR-Z decreased by 0.26 (95% CI 0.02 to 0.50). WC decreased by 2.61 cm (0.12 to 5.09) after 6 months, and by 2.25 cm (−1.58 to 6.09) after 12 months, but the decrease at study end was not significant relative to baseline (Table 2). For the non-operated group a small, but significant, increase in the BMI of 1.50 (95% CI −0.13 to 2.87*)* between baseline and 12 months was observed (Table 3). A significant linear increase in LBM over the 12-month period, with an increase of 2.87 kg (95% CI 1.47 to 4.26) and 2.32 kg (95% CI 0.40 to 4.23) at study end, was observed for both the operated and non-operated groups, respectively.

**Table 2:** Comparison of anthropometric, body composition, cardiorespiratory and cardio-metabolic risk factor outcome measures for the operated group.

Measurements	Baseline, mean (SD)	6 months, mean (SD)	12 months, mean (SD)	*p*-Value	Mean difference *B* to 6 (95% CI)	Mean difference *B* to 12 (95% CI)
Heart rate^b^ (bpm)	97.7 (10.1)	89.2 (16.9)	98.2 (12.6)	0.873	−8.4 (−19.2 to 2.4)	0.5 (7.7 to 8.7)
Systolic BP^a^ (mmHg)	114.0 (11.7)	114.8 (6.8)	116.4 (8.7)	0.225	0.8 (−5.4 to 7.0)	2.4 (−5.0 to 9.8)
Diastolic BP^a^ (mmHg)	62.9 (8.4)	68.3 (2.3)	69.9 (5.4)	0.404	5.3 (−1.0 to 11.6)	7.0 (−0.2 to 14.2)
BMI (kg/m^2^)	30.17 (4.95)	29.71 (4.47)	29.79 (4.31)	0.207	−0.47 (−1.50 to 0.57)	−0.38 (−1.79 to 1.02)
BMI-Z	2.06 (0.374)	1.95 (0.398)	1.92 (0.395)	0.012*	−0.10 (−0.21 to −0.002)*	−0.14 (−0.28 to −0.002)*
WC^a^ (cm)	95.91 (11.75)	93.30 (11.77)	93.65 (10.63)	0.028*	−2.61 (−5.09 to −0.12)*	−2.25 (−6.09 to 1.58)
WC-Z^a^	1.72 (0.393)	1.53 (0.546)	1.48 (0.551)	0.001*	−0.19 (−0.33 to −0.06)*	−0.24 (−0.43 to −0.05)*
WHtR	0.584 (0.076)	0.562 (0.084)	0.566 (0.096)	0.994	−0.022 (−0.039 to 0.004)	−0.024 (−0.049 to 0.002)
WHtR-Z	1.50 (0.574)	1.27 (0.719)	1.24 (0.718)	0.003*	−0.23 (−0.38 to −0.08)*	−0.26 (−0.50 to −0.02)*
Fat mass (kg)	33.28 (10.22)	32.02 (9.53)	32.27 (9.53)	0.177	−1.26 (−3.25 to 0.73)	−1.01 (−4.34 to 2.32)
Body fat (%)	41.08 (7.30)	38.64 (8.09)	38.24 (8.04)	0.297	−2.44 (−5.86 to 0.97)	−2.84 (−6.20 to 0.51)
Lean body mass (kg)	47.97 (13.92)	49.23 (14.18)	50.84 (13.94)	0.009*	1.26 (0.06 to 2.47)*	2.87 (1.47 to 4.26)**
Lean body mass (%)	57.13 (6.71)	58.44 (7.80)	59.01 (7.65)	0.731	1.31 (−0.81 to 2.69)	1.88 (0.30 to 4.06)

*p-value from RM-ANOVA or RM-ANCOVA*.

*95% CI, 95% confidence interval for mean difference between baseline (B) and 6 months (6) or 12 months (12); BP, blood pressure; BMI, body mass index; WC, waist circumference; WHtR, waist to height ratio; -Z, z-score*.

*n = 18, unless otherwise indicated*.

*^a^n = 16*.

*^b^n = 17*.

*Post hoc test p ≤ 0.05.

**Post hoc test p ≤ 0.001.

**Table 3:** Comparison of anthropometric, body composition, cardiorespiratory and cardio-metabolic risk factor outcome measures for the non-operated group.

Measurements	Baseline, mean (SD)	6 months, mean (SD)	12 months, mean (SD)	*p*-Value	Mean difference *B* to 6 (95% CI)	Mean difference *B* to 12 (95% CI)
Heart Rate^c^ (bpm)	102.7 (10.5)	96.8 (12.4)	102.2 (14.4)	0.910	−5.91 (−16.4 to 4.6)	−0.55 (−7.7 to 6.6)
Systolic BP^a^ (mmHg)	119.5 (8.2)	119.6 (10.3)	122.2 (9.9)	0.412	0.08 (−8.6 to 8.8)	2.7 (−5.9 to 11.3)
Diastolic BP^a^ (mmHg)	72.9 (8.4)	70.1 (6.3)	69.5 (6.6)	0.335	−2.8 (−11.7 to 6.2)	−3.3 (−11.7 to 5.1)
BMI (kg/m^2^)	27.74 (4.42)	27.95 (4.24)	29.24 (4.81)	0.046*	0.20 (−0.65 to 1.06)	1.50 (−0.13 to 2.87)*
BMI-Z	1.63 (0.49)	1.61 (0.52)	1.70 (0.55)	0.182	−0.026 (−0.16 to 0.11)	0.07 (−0.10 to 0.24)
WC^b^ (cm)	98.0 (13.5)	99.5 (8.5)	103.2 (11.8)	0.760	1.6 (−3.7 to 6.8)	5.3 (−0.3 to 10.3)
WC-Z^b^	1.56 (0.49)	1.62 (0.34)	1.67 (0.41)	0.312	0.07 (−0.14 to 0.26)	0.12 (−0.14 to 0.37)
WHtR^b^	0.60 (0.07)	0.60 (0.05)	0.62 (0.07)	0.892	0.003 (−0.025 to 0.032)	0.022 (−0.012 to 0.055)
WHtR-Z^b^	1.47 (0.52)	1.55 (0.33)	1.62 (0.44)	0.287	0.08 (−0.15 to 0.31)	0.15 (−0.18 to 0.48)
Fat Mass (kg)	29.8 (9.5)	30.1 (9.1)	32.7 (11.3)	0.076	0.23 (−2.93 to 3.39)	2.93 (−1.46 to 7.13)
Body Fat (%)	39.5 (5.5)	39.2 (5.7)	40.3 (6.5)	0.579	−0.29 (−2.7 to 21)	0.76 (−2.26 to 379)
Lean Body Mass (kg)	44.3 (86)	45.0 (84)	46.6 (8.5)	0.000*	0.73 (−1.12 to 2.59)	2.32 (0.40 to 4.23)*
Lean Body Mass (%)	59.3 (5.35)	58.8 (5.78)	57.8 (6.34)	0.220	−0.51 (−2.41 to 1.39)	−1.48 (−4.03 to 1.07)

*p-value from RM-ANOVA or RM-ANCOVA*.

*95% CI, 95% confidence interval for mean difference between baseline (B) and 6 months (6) or 12 months (12); BP, blood pressure; BMI, body mass index; WC, waist circumference; WHtR, waist to height ratio; -Z, z-score*.

*n = 14, unless otherwise indicated*.

*^a^n = 13*.

*^b^n = 12*.

*^c^n = 11*.

*Post hoc test p ≤ 0.05.

Examining the trend in BMI-Z prior to the start of the study until study completion revealed interesting trends (Figure 2). For the operated group (solid black line), the BMI-Z increased over time until the start of the intervention at which the BMI-Z showed a decline until the study end. However, for the non-operated group (solid gray line), the BMI-Z showed a similar increase over time prior to the start of the intervention, but leveled off after, and reverted back to an increasing trajectory. Of note, the BMI-Z was approximately 0.5 higher throughout in the operated group relative to the non-operated group. This is attributed to the group composition, as 74% of the operated, and 27% of the non-operated, group had obesity. A pre-intervention trajectory was determined for each group using linear regression on data spanning 42 months prior to, and including, baseline (dashed lines). Comparing the overall line of fit to the trajectories one can readily observe the impact of the intervention. The decrease in BMI-Z in the operated group is apparent, but also of note is the disruption to an expected increase in BMI-Z in the non-operated group (Figure 2).

## Discussion

Our pilot and feasibility study on a smartphone-based lifestyle intervention successfully demonstrated improved anthropometric and body composition measures in children with overweight or obesity and operated CHD. While we did not observe the same outcomes in the non-operated group, we did observe a disruption in the predicted linear increase in BMI-Z likely attributed to the intervention. To the best of our knowledge, this is the first study to implement this type of intervention in a pediatric CHD population. Furthermore, our study had superior participant retention and attrition rates compared to similar lifestyle intervention studies (22). We believe this to be attributed to the use of smartphones in the study, which enabled us to effectively engage with the participants, despite geographic or scheduling limitations. Overall, the results from this study effectively demonstrated the feasibility of the current protocol, and the implemented remote lifestyle intervention strategy, to move forward with a larger controlled study.

In a systematic review of 38 eligible studies conducted between 1975 and 2010, Ho et al. concluded that pooled pediatric obesity lifestyle interventions reduced BMI-Z by 0.10 (95% CI 0.02–0.18) (22). A similar change was seen in the operated study group, suggesting the intervention was as successful as other interventions at decreasing and sustaining BMI-Z for the 6- and 12-month periods. Few lifestyle intervention studies on children and adolescents with overweight or obesity employed WC, WHtR, or their corresponding *z*-scores as part of their outcome measures; for studies that did so, the results were inconsistent (22).

For the operated group, changes in BMI-Z, WC, WC-Z, and WHtR-Z occurred within the first 6 months of the program and remained relatively unchanged at 12 months. This was not unexpected, as Franz et al. reported that the largest loss in weight occurred within the first 6 months and was maintained after 12 months in a review of 80 weight-loss intervention studies in adults (23). Moreover, Franz et al. stated that although weight loss leveled off after the initial reduction, stopping the interventions altogether would likely have led to weight gain (23). Since there were no increases in anthropometric measures after the 6-month point in our study, this suggests that participants in the operated group were likely still engaged with the intervention while the non-operated group was not. This also indicates that the intervention may not have been rigorous enough, and positive changes may have continued with a more aggressive diet and exercise plan.

The significant findings in the operated group were not entirely replicated in the non-operated group, although they both received the identical intervention. We believe this may be due self-efficacy and how the parents/patients perceive the seriousness of their condition in relation to lifestyle (24–26). For example, the operated group may have had a much better understanding of their disease and the impact of overweight and obesity on their health and were more engaged as a result. It is also possible the operated group had been more reserved in their physical activity due to the perceived seriousness of their condition and increased efforts during the intervention as it was regularly monitored by medical professionals. However, it is important to note that most of the participants in the operated group were obese, whereas the non-operated participants were predominantly overweight, which may have enabled more rapid positive changes from the intervention. We believe further studies are warranted to test these hypotheses.

A meta-analysis by Stoner et al. found that structured exercise interventions slightly increased LBM by 1.6 kg (95% CI 0.5–2.6) while also attenuating percent body fat (%BF) by 3.1% (95% CI 2.2–4.1) in overweight and obese adolescents (27). While we observed a significant linear increase in age and sex adjusted LBM by 2.9 kg (95% CI 1.47–4.26) from baseline, there was no apparent increase in the percent LBM. This suggests that LBM and total body mass increased linearly together over the 12-month period. However, there appeared to be decreases in the fat mass and %BF from baseline to 6 and 12 months in our study, but it did not reach statistical significance due to the large variance (Table 2). Thus, the increased LBM is likely the result of normal childhood growth. This is not surprising given that our intervention did not include a regimented physical exercise component and structured resistance or weight training components. Also, another possibility was the sample size in our study was not representative enough to adjust for the strong association between LBM and pubertal growth.

We did not observe any significant changes in any of the measured cardio-metabolic outcomes in our study as reported with other interventions (22). However, as reported in a review by Ho et al., there were no clear associations between the extent of weight loss or body fat reduction and improvements in cardio-metabolic outcomes (22). Thus, the changes observed in these studies may independently result from the lifestyle intervention itself, through increased physical activity and an improved diet, as opposed to weight loss directly. While we did observe positive anthropometric changes in our study, the intervention may not have been adequate enough to drive improvements in cardio-metabolic outcomes.

A significant increase in peak VO_2_ has been reported in several studies on physical exercise training programs in children and young adults with CHD (28, 29). Similar age and sex adjusted results were not observed in our study, which was likely a consequence of lacking a regimented physical exercise component and participant engagement.

The differences observed between this study and those reported in the reviews by Ho et al. (22), Tikkanen et al. (28), and Duppen et al. (29) may be explained by limitations of the intervention components. For instance, Ho et al. (22) noted the features of effective interventions were: family involvement, dietary intervention (typically a structured restrictive diet), and structured exercise training (22). Unfortunately, the Smart Heart Pilot Study did not strive for significant family involvement, which may have had an impact on the study, as engaging family members can enhance the effectiveness of weight management (30, 31). Also, dietary and fitness components were designed to be realistic, sustainable, and empowering for young people living in an uncontrolled environment; as such we did not prescribe regimented diet and exercise plans. Since the physical activity component of lifestyle interventions is specifically associated with improvements in body composition, one must also consider the differing degrees of physical activity participation (frequency, duration, and intensity) achieved by study participants as a potential explanation. Furthermore, reliance on participant self-report regarding their engagement in physical activity was an additional challenge to increasing physical activity levels in this remote intervention as self-report can be influenced by social desirability and recall bias, and may also lead to overestimations of both the quantity and intensity of effort (32).

Ultimately, one of the primary reasons we did not observe significant differences in many of the cardio-metabolic and body composition outcome measures was likely a consequence of the small sample size used in the study. We were hopeful the intervention-driven changes would be large enough to detect, but unfortunately the power of the study was too low to detect these small differences. This was also compounded by the large degree of variation in the metrics used given the age range of the study population and the large rapid changes associated with puberty. This can be particularly true for changes in height, body fat distribution, and insulin levels over a 12-month period. Furthermore, our primary interest was to determine if the proposed remote-counseling intervention would be feasible in a pediatric CHD population, which our results support. However, not including a control non-CHD population has introduced some limitations to the study interpretation regarding the presence of CHD and its influence on patient engagement and measured outcomes. Ideally, a randomized control trial would prove to be most beneficial and believe our current protocol is applicable to a larger randomized control study.

Retention is also an important indicator of program feasibility. A retention rate of 100% (i.e., no drop-outs) and an attrition rate of 2% (i.e., 2 of 102 clinic visits missed) are atypical for a lifestyle intervention program of this duration and nature. Dhaliwal et al. documented a median attrition rate of 37% (range 4–83%) from 23 published pediatric lifestyle interventions (33). Logistical barriers were one of the most commonly cited reasons for dropout. The Smart Heart Pilot Study reduced the need for families to travel during the intervention by providing remote counseling *via* smartphones. The majority of participants would have been required to travel ~50 to 200 km to the hospital. Smartphone counseling removed the burdens of traveling to the hospital, missed school and work, as well as the costs of public transit, petrol, and parking. It is also important to note that enrolled subjects may have been more amenable to lifestyle changes simply due to their interest in participating in the study, which could lead to bias in the results.

There is no research evaluating lifestyle interventions for children with overweight or obesity and CHD using smartphones. This is the first study to explore this novel approach to implementing a lifestyle intervention in this high risk population. The Smart Heart Pilot Study proved to be cost-effective and demonstrated a superior participant retention rate compared to similar pediatric lifestyle interventions not using smartphones for remote counseling. Preliminary evidence demonstrates that the Smart Heart Pilot Study had a positive effect on LBM, BMI-Z, WC, WC-Z, and WHtR-Z, but this may have been dependent on self-efficacy and the perception of the underlying heart condition. Despite some study limitations, the results suggest that a larger study would be highly feasible and likely prove more successful with improvements to the intervention protocol. Continued research should elucidate whether the aforementioned enhancements to the delivery of pediatric obesity lifestyle interventions demonstrate further improved cardiovascular health outcomes.

## Ethics Statement

This study was carried out in accordance with the recommendations of the Western University, Lawson Health Research Institute and London Health Sciences Centre research guidelines with written informed consent from all subjects. All subjects gave written informed consent in accordance with the Declaration of Helsinki. All children and adolescents gave written informed consent to participate in this study. For those under 16 years of age, we obtained written informed consent from their parents/guardians. The protocol was approved by the Western University Health Science Research Ethics Board (REB#18843) and the Lawson Health Research Institute (R-12-266).

## Author Contributions

LA-D is a pediatric cardiologist at London Health Sciences Centre (LHSC) and ran clinics for the study. MR is a nutritionist and provided nutrition counseling for the study and contributed to the manuscript. SJ is an exercise and health psychology researcher and provided physical activity counseling for the study and helped design the study. EW is a pediatric cardiologist at LHSC and ran clinics for the study and helped design the study. HP is the Director of the Exercise and Health Psychology Laboratory at Western University and helped design the study and contributed to the manuscript. DF is pediatrician and head of Translational Research Centre and was responsible for lab work and contributed to the manuscript. AAD is a research assistant at LHSC and contributed to statistical analysis and manuscript preparation. MRM is a statistician and provided statistical analysis for the study. KN is a pediatric cardiologist at LHSC and ran clinics for the study, and was the sponsor and principal investigator for the study.

## Conflict of Interest Statement

The authors declare that the research was conducted in the absence of any commercial or financial relationships that could be construed as a potential conflict of interest.

## Abbreviations

CHD, congenital heart disease; iDXA, dual X-ray absorptiometry; BMI, body mass index; BMI-Z, body mass index *z*-score; WC, waist circumference; WC-Z, waist circumference *z*-score; WHtR, waist to height ratio; WHtR-Z, waist to height ratio *z*-score; FM, fat mass; BF, body fat; %BF, percent body fat; LBM, lean body mass; VO_2max_, maximal volume oxygen uptake; LDL, low density lipoproteins; HDL, high density lipoproteins; TG, triglycerides; HOMA-IR, homeostatic model assessment for insulin resistance.
